# Diagnostic and therapeutic journey of infantile endobronchial tuberculosis: a case report

**DOI:** 10.3389/fped.2026.1778717

**Published:** 2026-03-09

**Authors:** Zhi Li, Yi Zhang, Deyong Xu, Bo Huang

**Affiliations:** 1 Department of Pediatrics, Affiliated Hospital of Zunyi Medical University, Zunyi, Guizhou, China; 2 Department of Pediatrics, Guizhou Provincial Children’s Hospital, Zunyi, Guizhou, China; 3 Department of Pediatrics, Zunyi Medical University, Zunyi, Guizhou, China; 4 Department of Pediatrics, Zunyi Medical and Pharmaceutical College, Zunyi, Guizhou, China

**Keywords:** bronchoscopy, endobronchial tuberculosis, *IKZF1* mutation, infant, pneumocystis *jirovecii*, primary immunodeficiency

## Abstract

**Background:**

Endobronchial tuberculosis (EBTB) in infants is rare and is often overlooked because of nonspecific clinical manifestations. Coexisting primary immunodeficiency and opportunistic infections further increased diagnostic and therapeutic complexity.

**Case presentation:**

We reported a male infant aged 40 days who presented with fever and mild cough. Chest imaging showed progressive bilateral nodular and granulomatous lesions despite broad-spectrum antibacterial therapy. Microbiological evaluation revealed positive T-SPOT.TB and GeneXpert MTB/RIF results from bronchoalveolar lavage fluid (BALF), while metagenomic next-generation sequencing identified *Pneumocystis jirovecii*. Genetic testing demonstrated a heterozygous *IKZF1* mutation, consistent with underlying immunodeficiency. Serial bronchoscopies confirmed necrotizing endobronchial tuberculosis with airway stenosis. The patient received standard anti-tuberculosis therapy, systemic corticosteroids, trimethoprim–sulfamethoxazole, intravenous immunoglobulin, and repeated bronchoscopic intraluminal drug delivery. Clinical and radiological remission was achieved, with no airway sequelae during 18-month follow-up.

**Conclusions:**

This case highlighted the unique coexistence of infantile EBTB, *IKZF1*-related immunodeficiency, and *P. jirovecii* coinfection. Early bronchoscopy played a pivotal diagnostic and therapeutic role. Repeated intraluminal bronchoscopic therapy combined with systemic treatment might prevent irreversible airway damage in severe pediatric EBTB.

## Introduction

### Background

Tuberculosis (TB) remains a major global cause of morbidity and mortality in children, particularly in infants who often present with atypical and nonspecific symptoms. Diagnostic confirmation is challenging because of low bacillary load and limited sputum production. Endobronchial tuberculosis (EBTB), defined as infection of the tracheobronchial tree, is an important but underrecognized manifestation of pediatric TB and may lead to airway stenosis, atelectasis, and long-term pulmonary sequelae if diagnosis is delayed (1–10).

Primary immunodeficiencies significantly increase susceptibility to severe and disseminated TB. *IKZF1* encodes the transcription factor Ikaros, which is essential for lymphoid development and immune regulation. Mutations in *IKZF1* result in combined humoral and cellular immune dysfunction, impairing host defense against intracellular pathogens such as *Mycobacterium tuberculosis*, and predisposing patients to severe infections and opportunistic pathogens, including *Pneumocystis jirovecii*.

We describe a rare and complex case of infantile pulmonary TB with EBTB, *P. jirovecii* co-infection, and *IKZF1*-related immunodeficiency, emphasizing the importance of early bronchoscopy and multidisciplinary management.

## Case presentation

The detailed timeline of the clinical course is summarized in Table 1.

**Table 1:** Timeline of the clinical course.

Date	Age (months)	Key clinical events
July 2023	1	Fever and cough; initial chest CT **showed** bilateral nodular lesions
July 2023	1	First bronchoscopy; BALF was positive for *Mycobacterium tuberculosis* and *Pneumocystis jirovecii*
August 2023	2	**Anti-tuberculosis** therapy was initiated, with temporary clinical improvement
August–September 2023	2–3	Clinical relapse occurred; chest CT **showed** airway obstruction and emphysema
September–November 2023	3–5	Four bronchoscopic interventions with intraluminal therapy were performed
February 2024	8	Bronchoscopy **confirmed** resolution of airway stenosis
January 2025	18	Chest CT **showed** complete lung **re-expansion**; treatment was completed

The bold font in **Table 1** highlights key clinical milestones during the disease course.

### Initial presentation and imaging strategy

A 40-day-old male infant presented with a 4-day history of fever and a 2-day history of mild cough. Given persistent fever, elevated inflammatory markers, and concern for severe lower respiratory pathology in a young infant, chest CT was performed early to better characterize parenchymal disease. (Chest radiography was not performed initially due to rapid clinical progression and the need for higher diagnostic sensitivity.)

The relevant imaging findings are shown in Figures 1–11.

**Figure 1:**
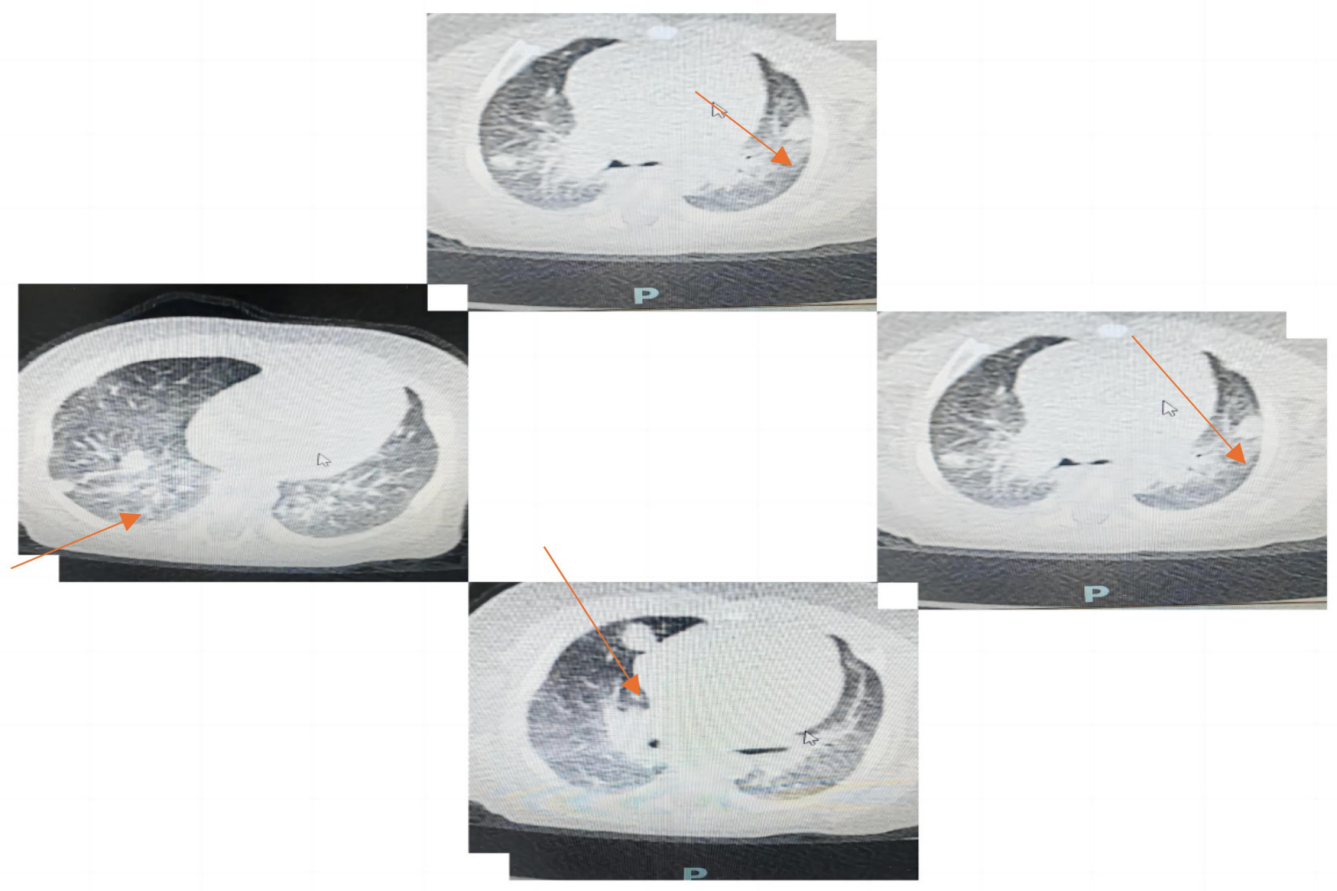
Initial chest computed tomography (CT) performed on July 13, 2023 showed multiple bilateral nodular and mass-like pulmonary lesions.

**Figure 2:**
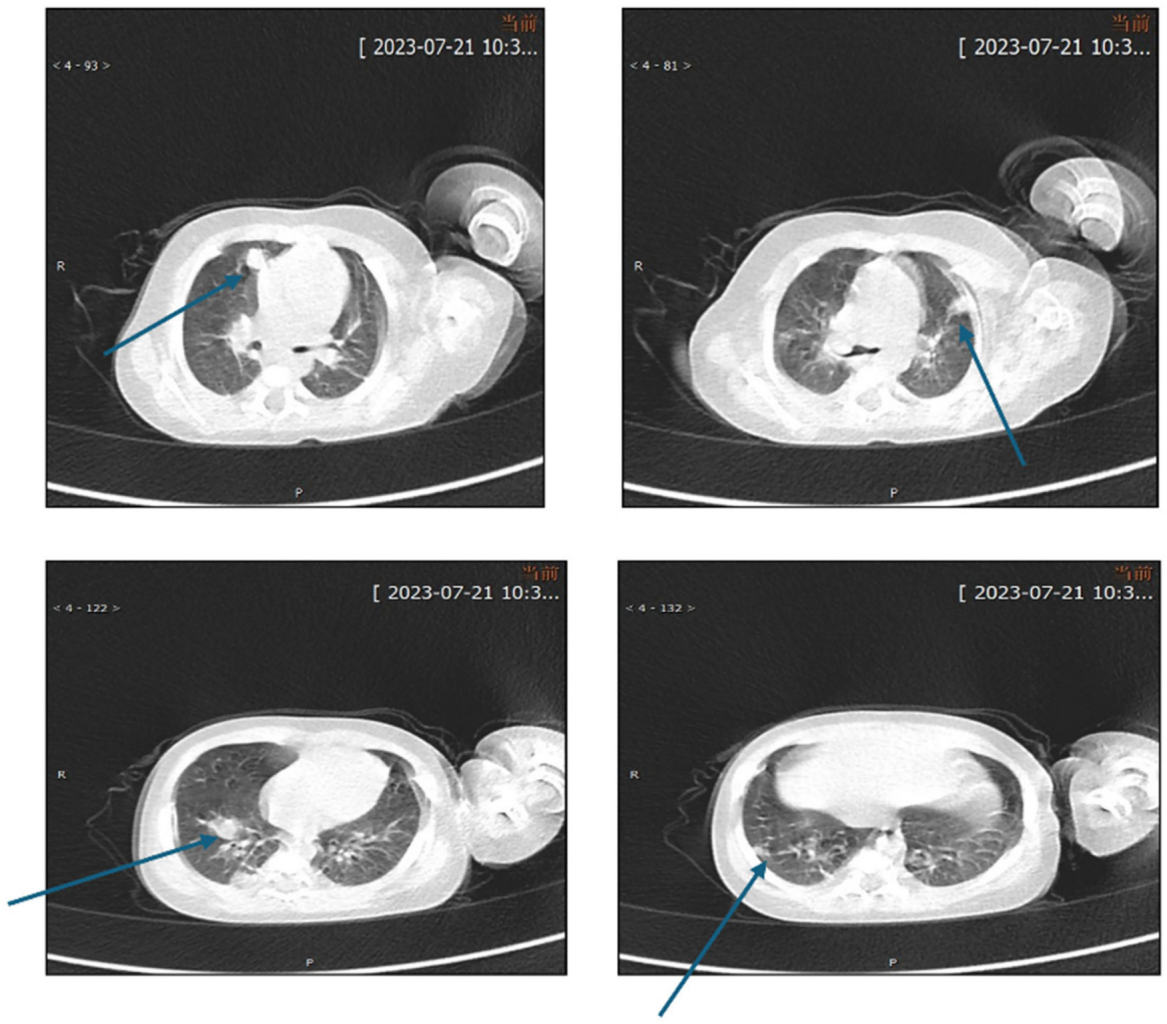
Chest CT obtained after 1 week of treatment showed partial radiological improvement of pulmonary lesions.

**Figure 3:**
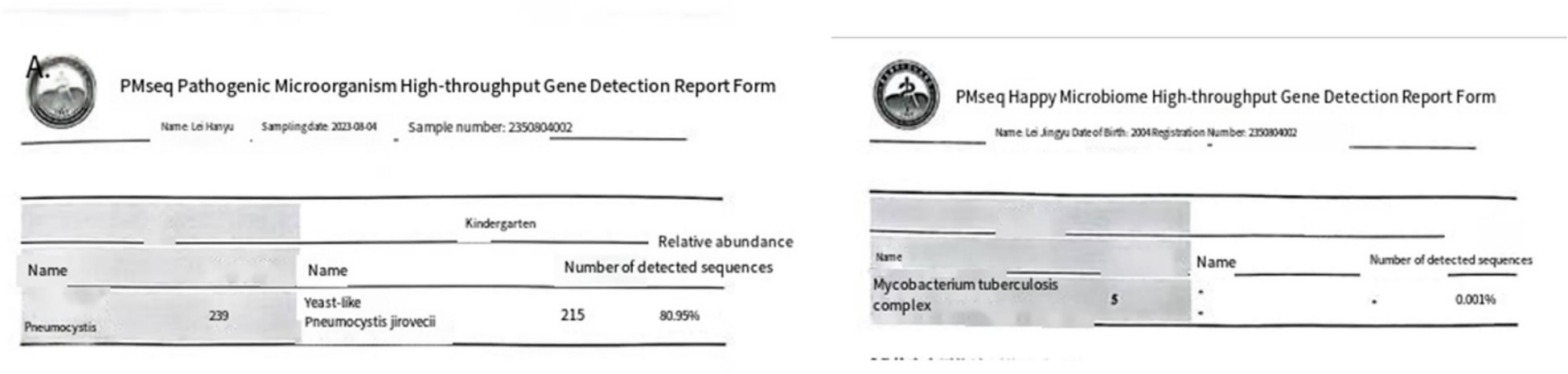
Metagenomic next-generation sequencing of bronchoalveolar lavage fluid identified *pneumocystis jirovecii* and *Mycobacterium tuberculosis* complex.

**Figure 4:**
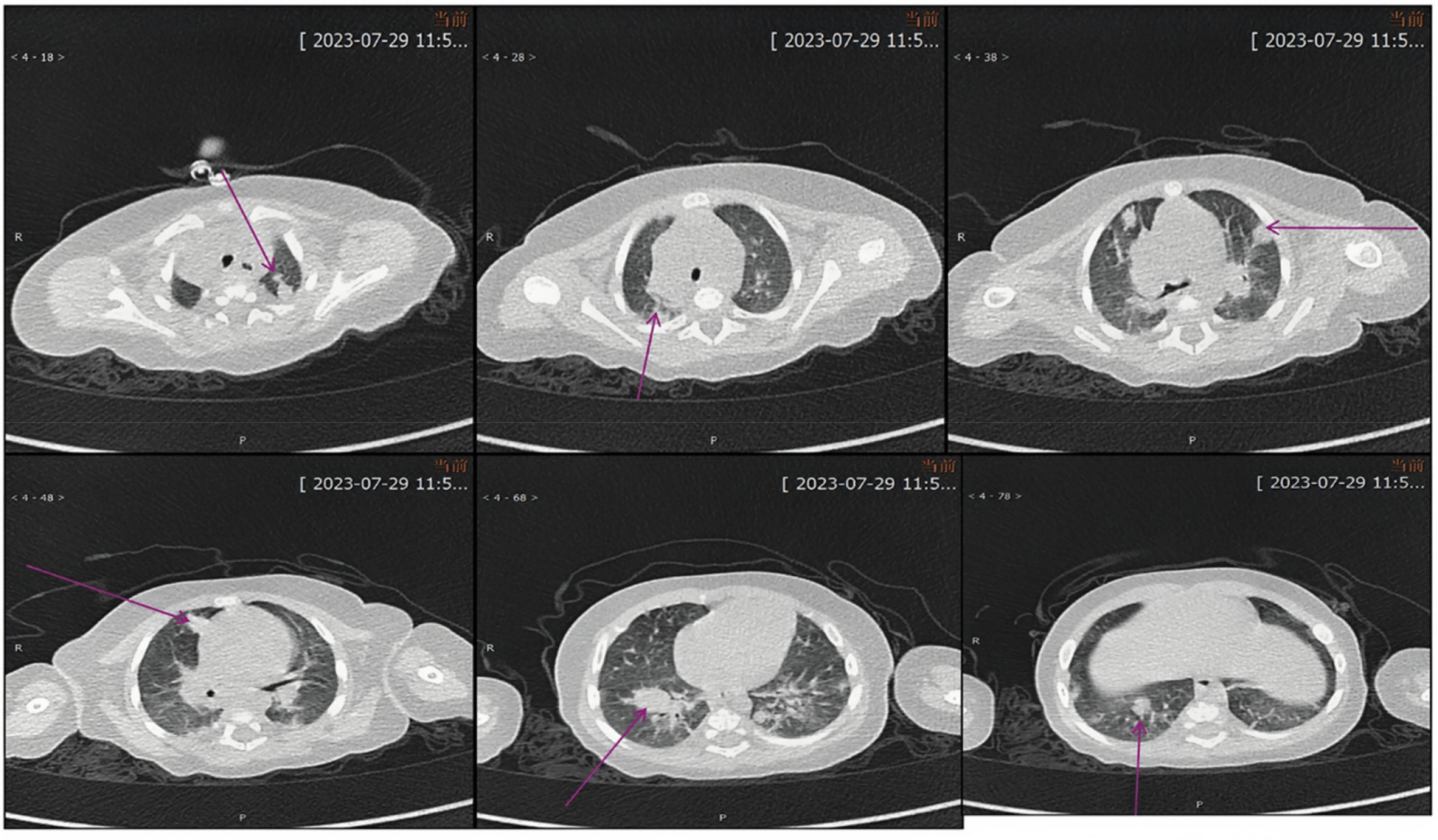
Contrast-enhanced chest CT performed on July 29, 2023 demonstrated multiple pulmonary lesions accompanied by mediastinal lymphadenopathy.

**Figure 5:**
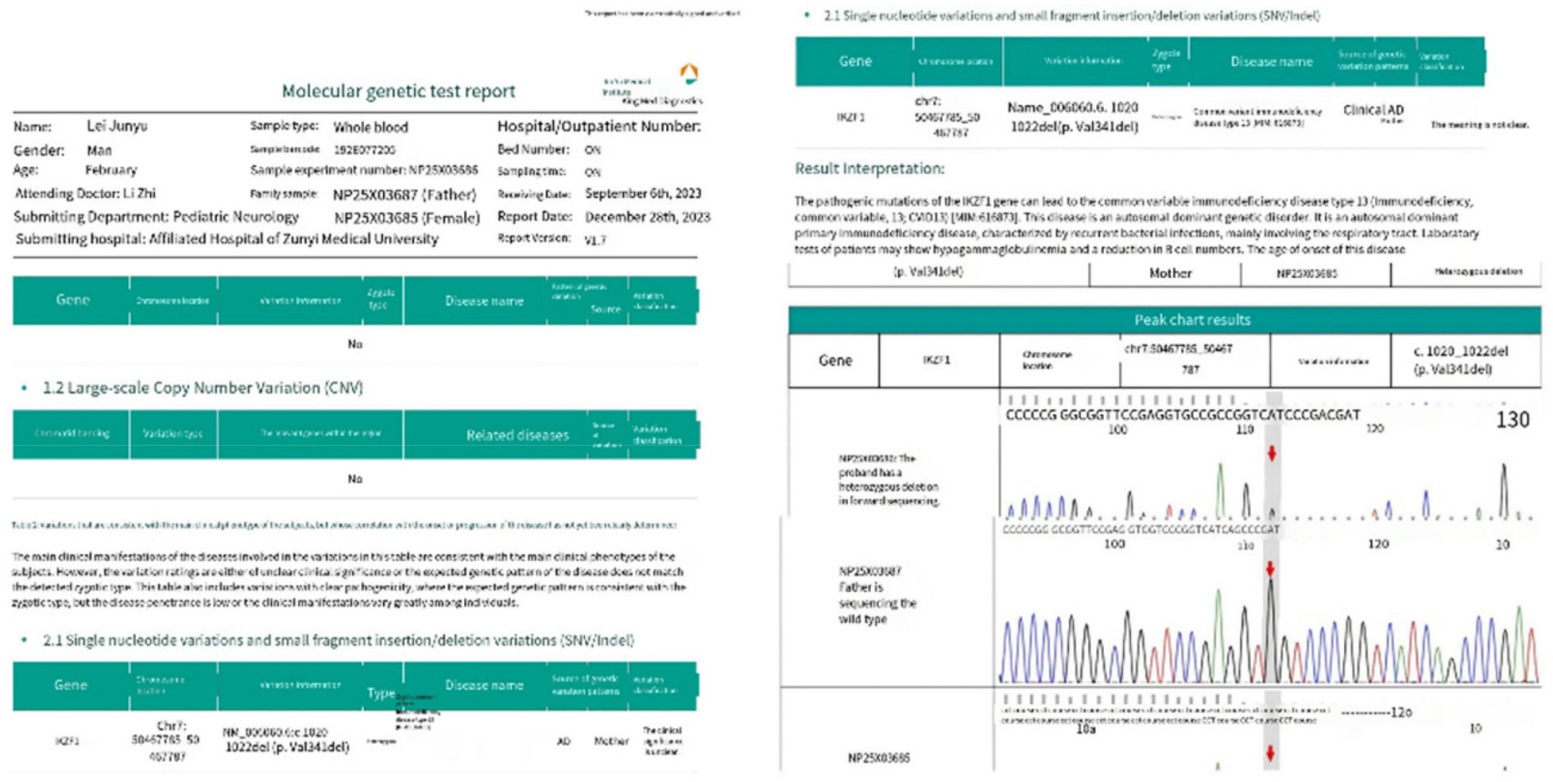
Genetic analysis revealed a heterozygous *IKZF1* mutation associated with underlying primary immunodeficiency.

**Figure 6:**
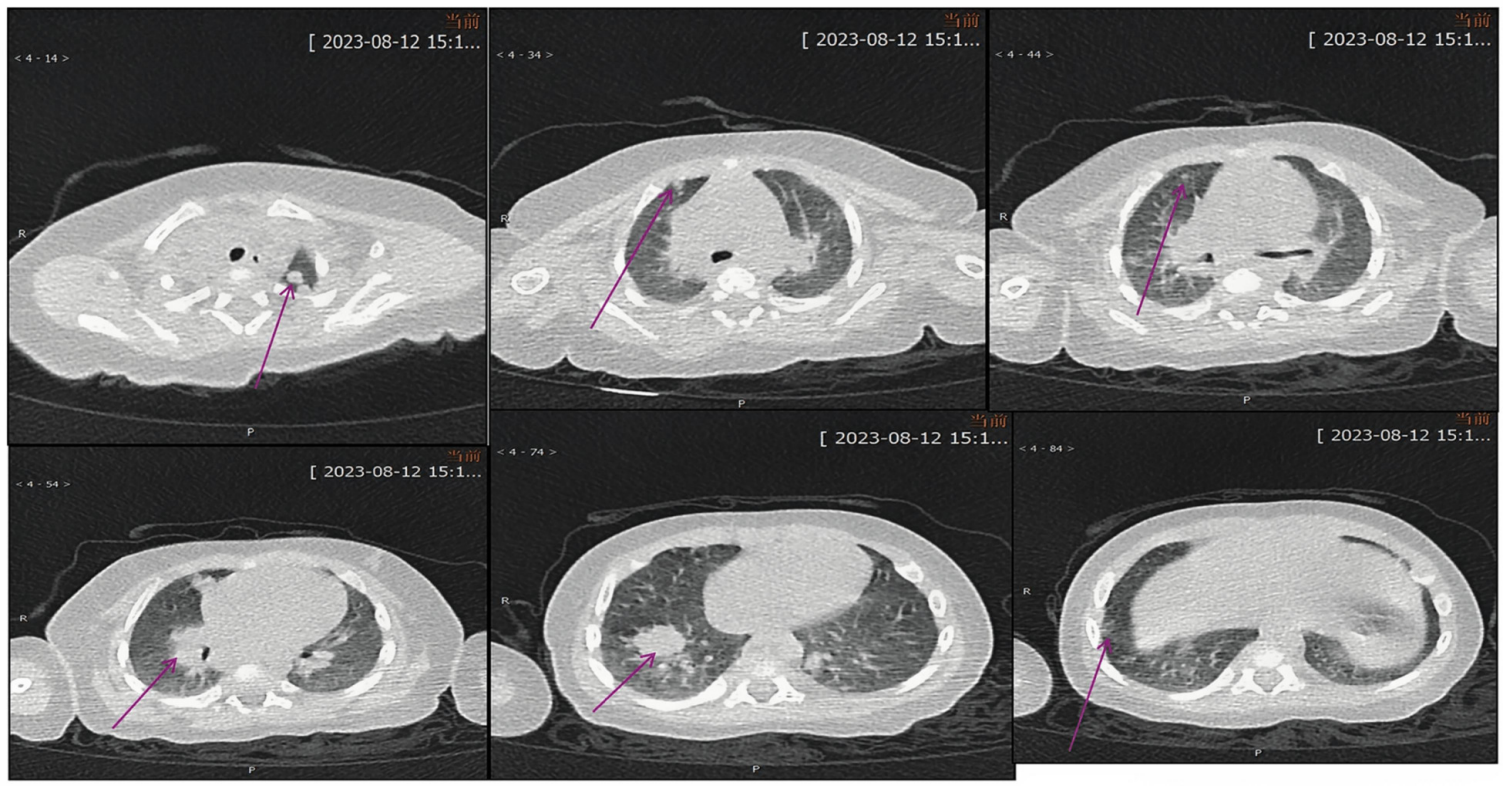
Chest CT prior to first hospital discharge on August 12, 2023 showed partial resolution of pulmonary lesions.

**Figure 7:**
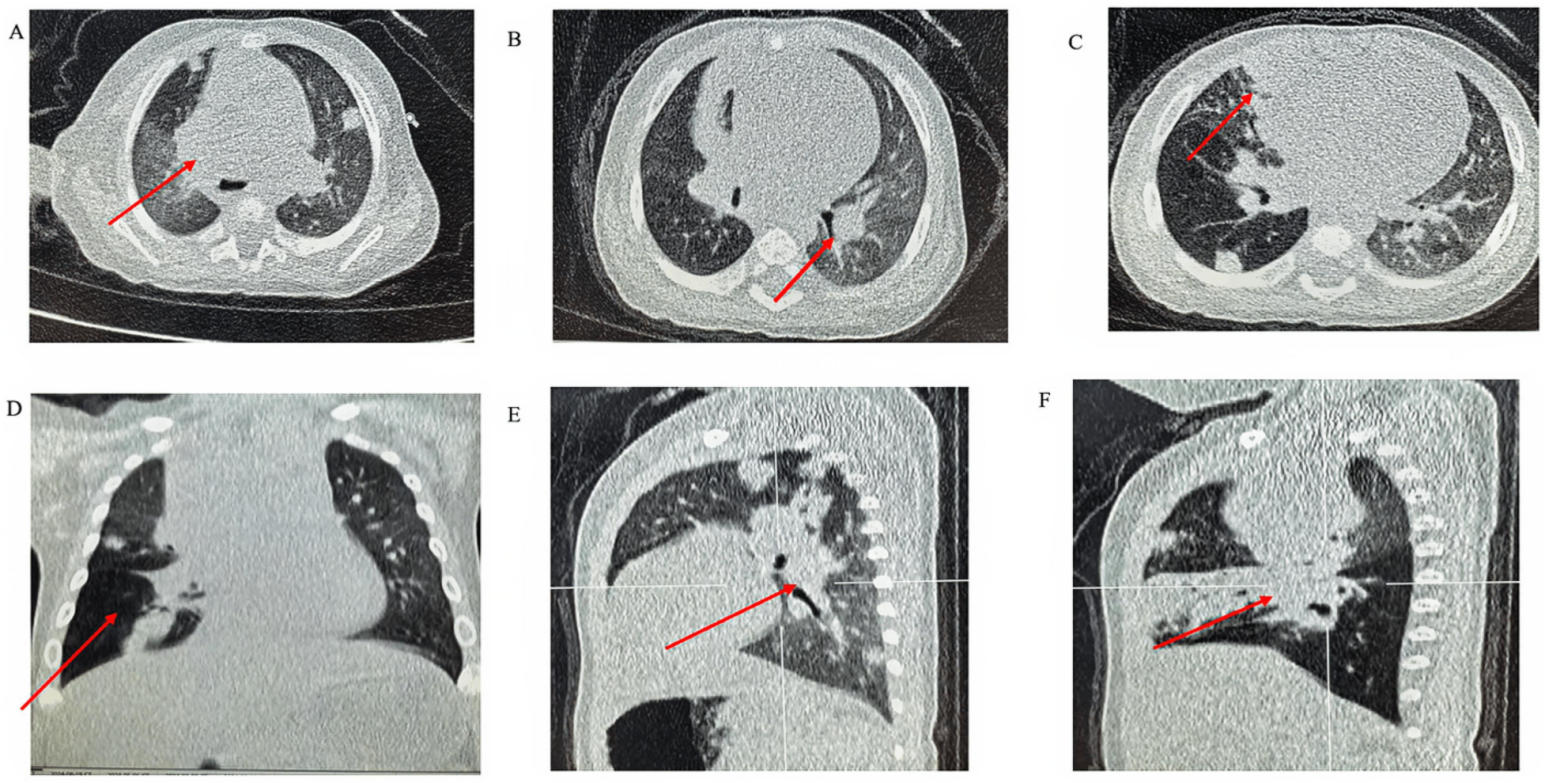
**(A–C)** Axial CT views demonstrating airway obstruction. **(D–F)** Coronal and sagittal reconstructions showing emphysematous changes and persistent nodular lesions.

**Figure 8:**
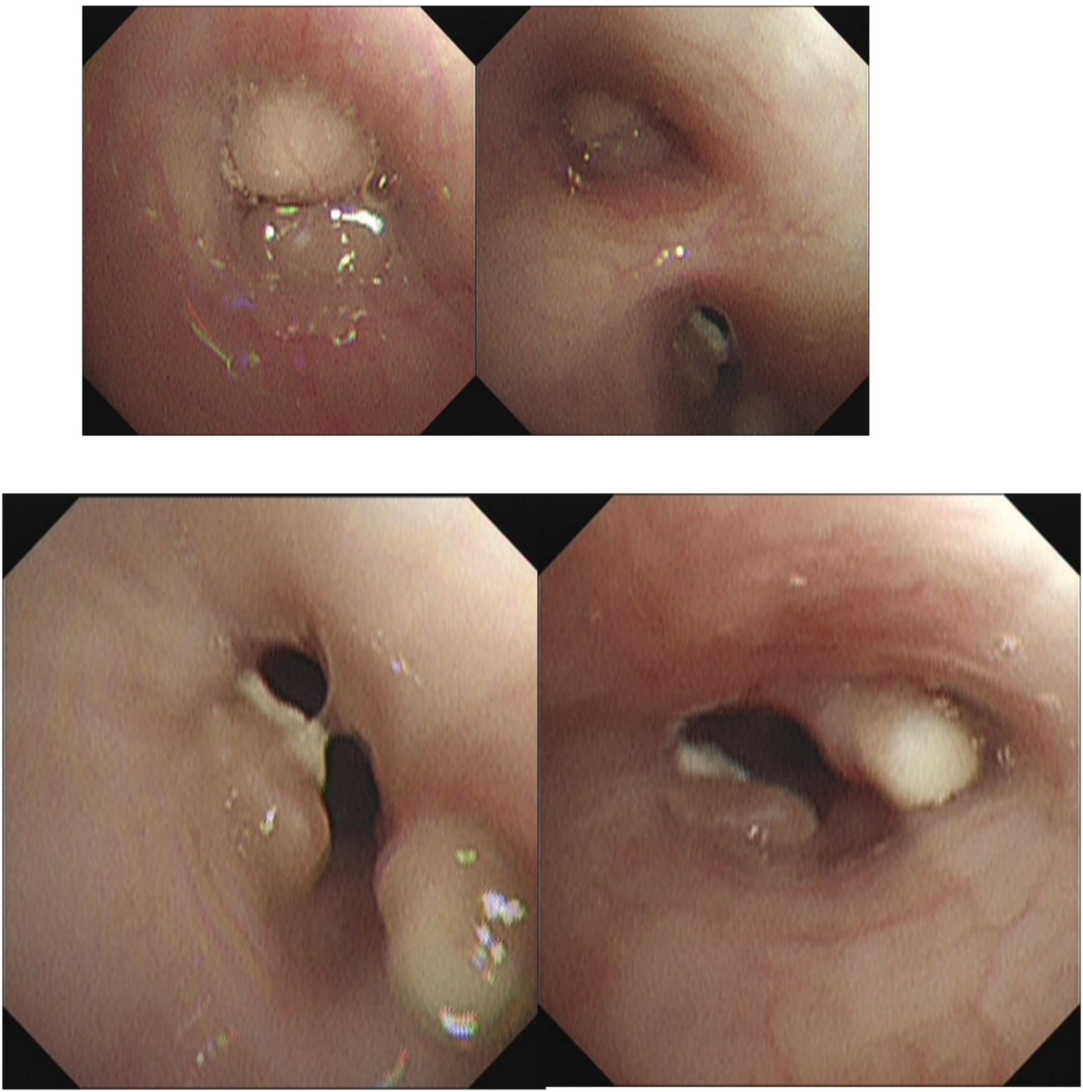


**Figure:**
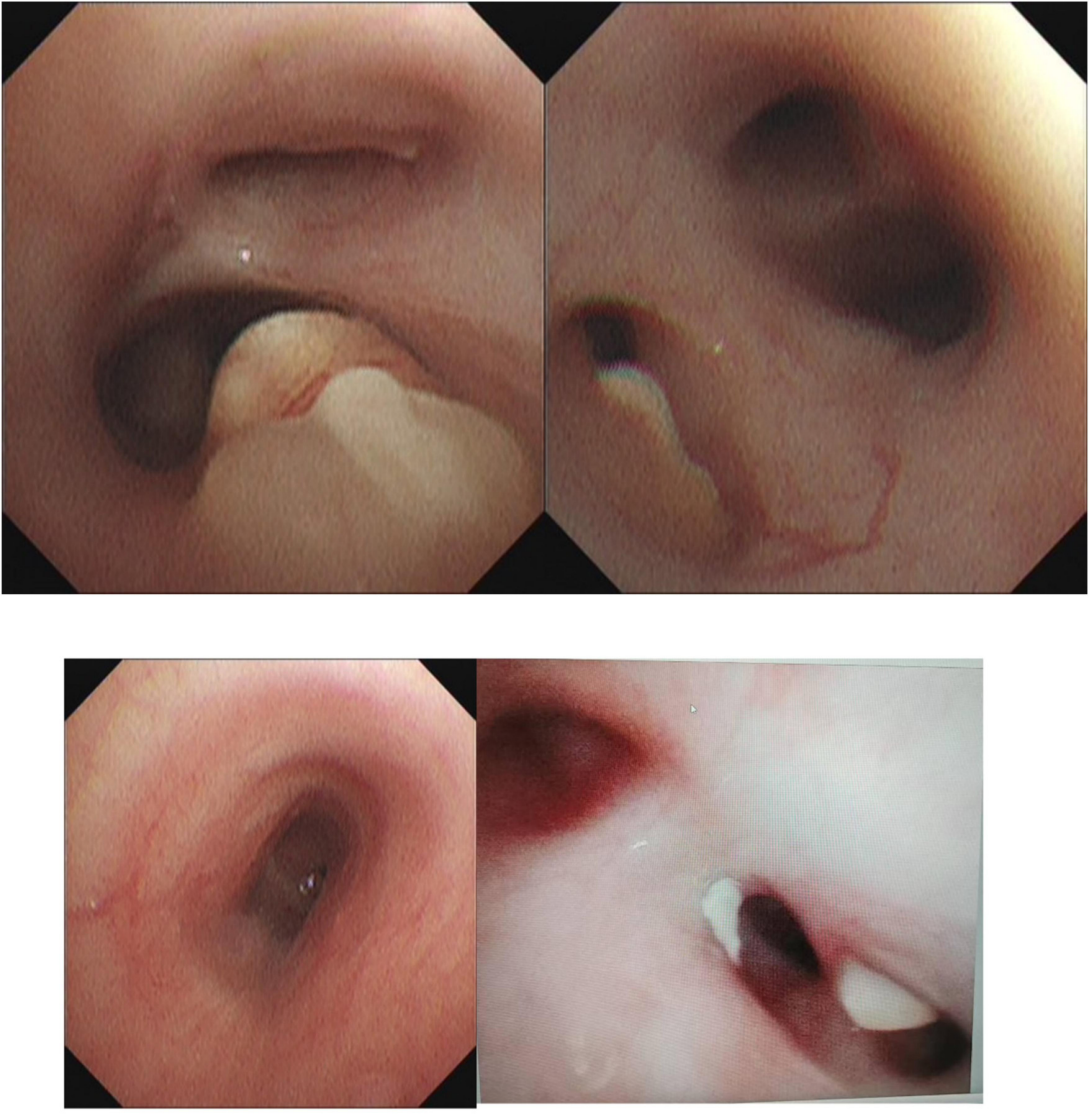
Bronchoscopic findings during four therapeutic interventions. Initial bronchoscopy showed necrotizing endobronchial lesions with luminal narrowing. Sequential bronchoscopies demonstrated progressive clearance of caseous material and gradual improvement of airway patency following intraluminal therapy.

**Figure 9:**
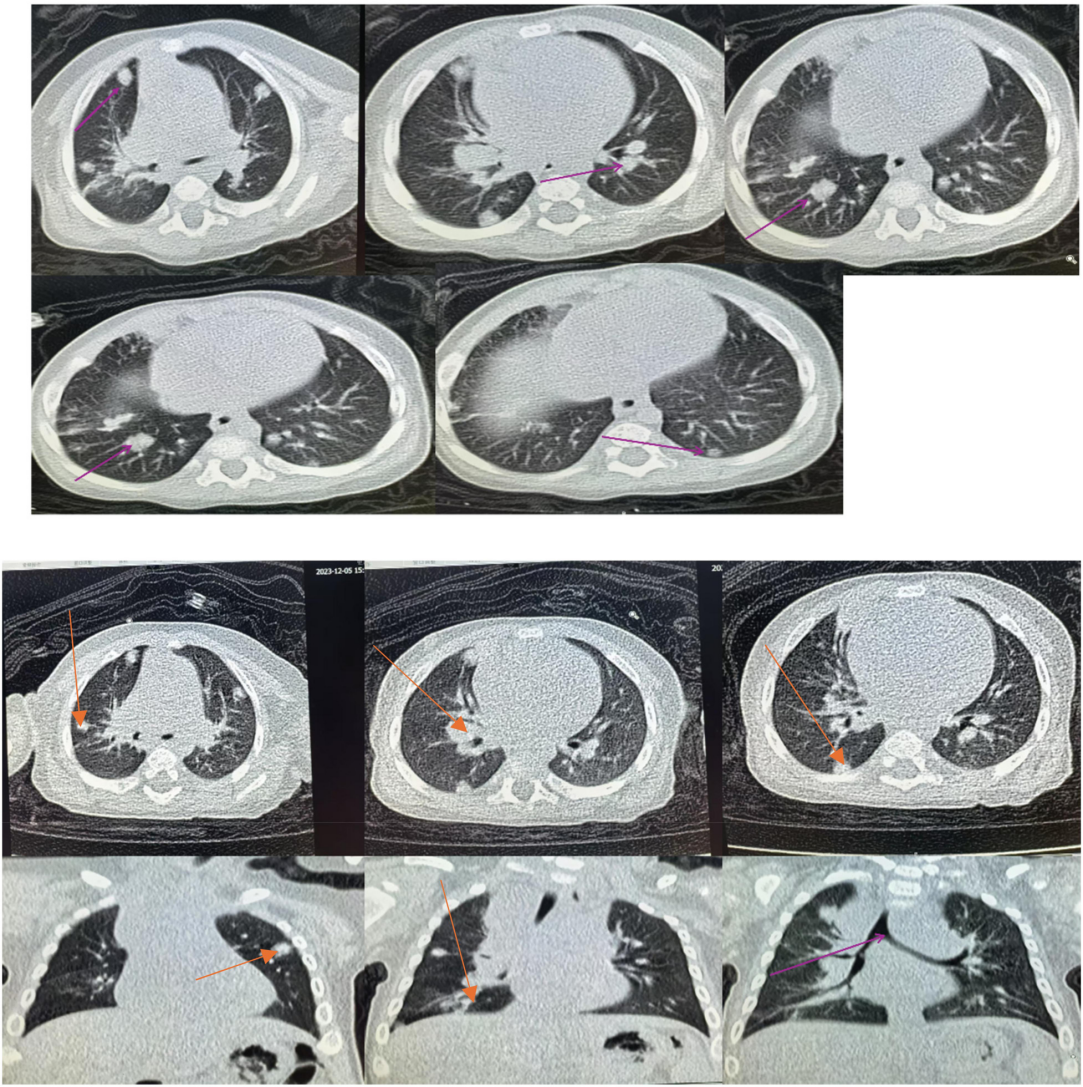
Serial chest CT images showed progressive improvement in airway obstruction and pulmonary nodular lesions following combined systemic and bronchoscopic treatment.

**Figure 10:**
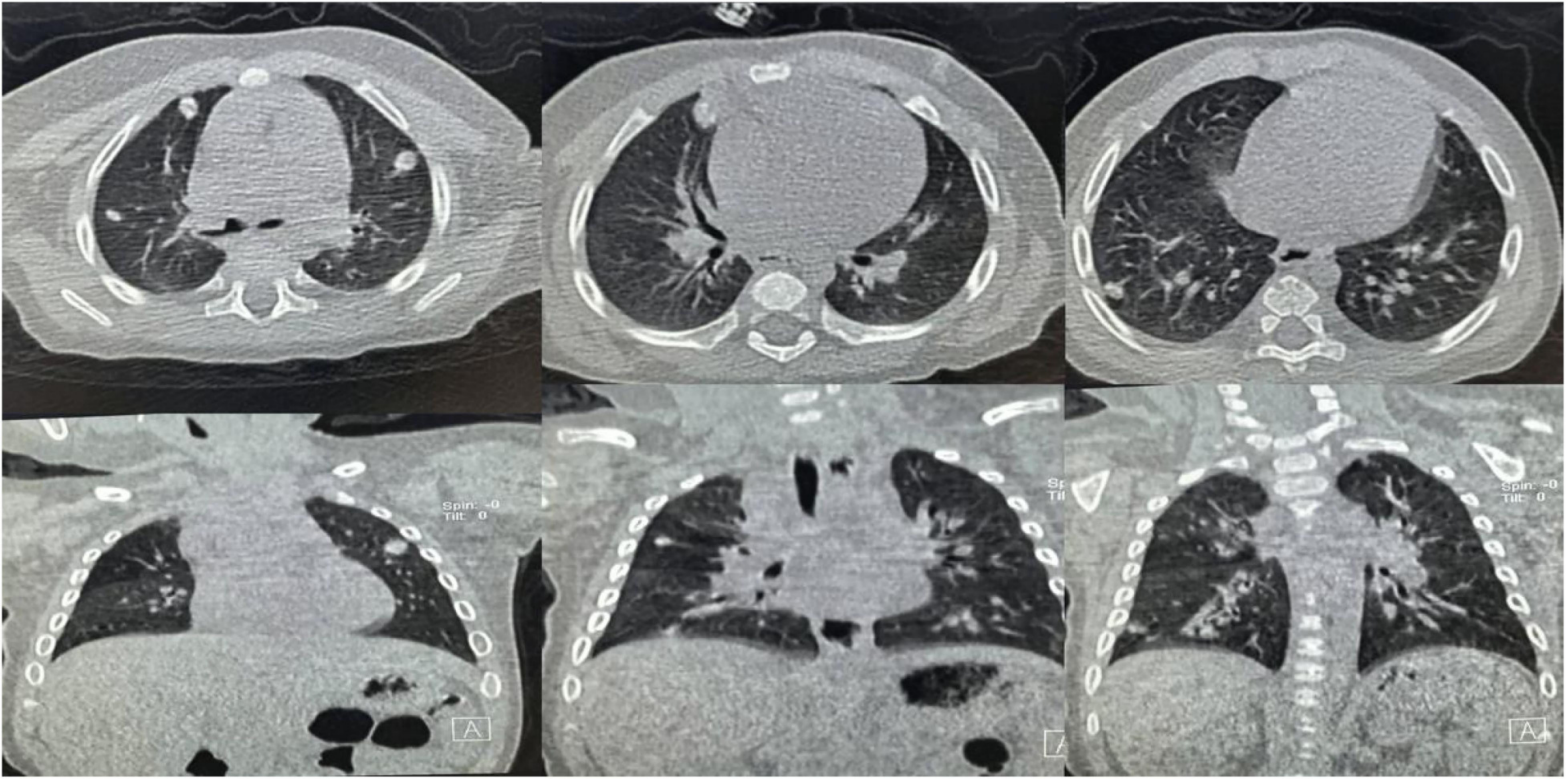
Bronchoscopy performed on February 19, 2024 confirmed resolution of endobronchial stenosis.

**Figure 11:**
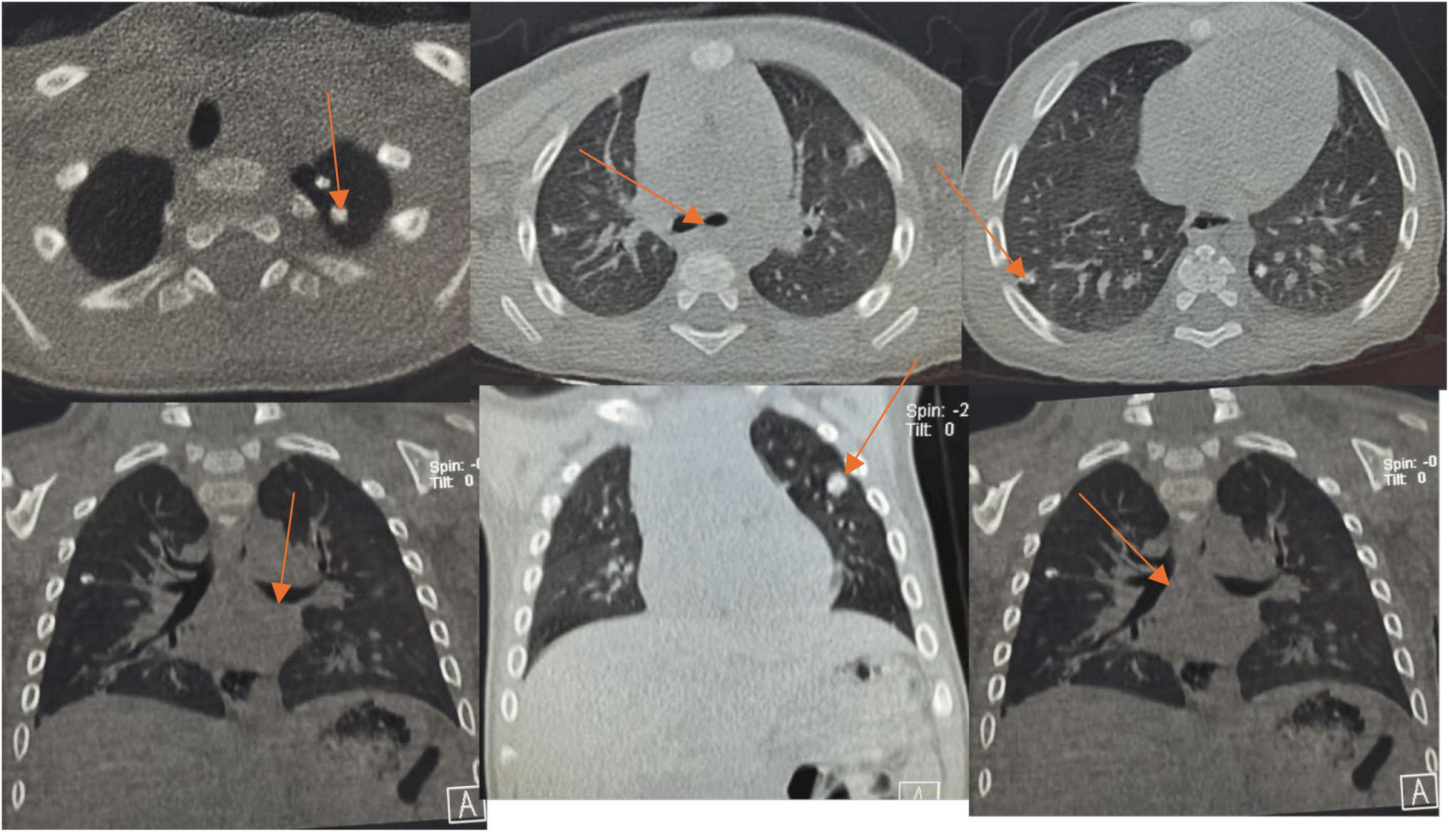
Chest CT obtained on January 17, 2025 showed complete lung re-expansion with residual calcified nodules.

### Initial management and rationale for antibiotics

Empirical antibacterial therapy was started. Linezolid was selected in combination with first-line anti-tuberculosis drugs because of concern for severe pneumonia, possible resistant Gram-positive infection, and the need for good lung tissue penetration while awaiting definitive microbiological results.

### Diagnostic workup

Bronchoscopy initially revealed diffuse bilateral airway inflammation without visible caseous lesions. Bronchoalveolar lavage fluid (BALF) analysis demonstrated positive T-SPOT.TB results on two occasions and a positive GeneXpert MTB/RIF assay, with no evidence of rifampicin resistance. Metagenomic next-generation sequencing of BALF identified *Pneumocystis jirovecii*, indicating opportunistic coinfection. Drug-resistant tuberculosis was carefully evaluated, and the microbiological findings, together with the clinical response, were consistent with drug-susceptible tuberculosis.

Genetic testing subsequently revealed a heterozygous *IKZF1* mutation, providing an explanation for the severity of disease and the patient's susceptibility to opportunistic infection.

Bronchoscopy was not performed at the earliest stage because the initial clinical presentation and imaging findings were nonspecific, and the patient's young age required careful consideration of procedural risks. As the disease progressed and airway involvement became more evident, bronchoscopy was subsequently performed and proved to be critical for both diagnosis and therapeutic management.

### Disease progression despite anti-TB therapy

Although anti-tuberculosis therapy was initiated promptly after microbiological confirmation, the patient's clinical course showed fluctuating progression during the early stage of treatment. This progression was likely multifactorial and was associated with the initially nonspecific clinical presentation, a high local inflammatory burden within the airways, airway obstruction caused by necrotizing endobronchial lesions, and the presence of underlying immunodeficiency. Systemic anti-tuberculosis therapy alone appeared insufficient to adequately control the localized endobronchial disease during this phase.

During this period, bronchodilator therapy was administered to relieve symptoms related to airway obstruction. However, medical management alone did not result in sustained improvement until targeted bronchoscopic evaluation and intervention were undertaken, after which clinical stabilization was gradually achieved.

### Respiratory status and bronchodilator therapy

During the acute phase of hospitalization, the patient's respiratory status was closely monitored. Although intermittent tachypnea and mild increased work of breathing were observed during disease exacerbations, there was no clinical evidence of respiratory failure.

Arterial or capillary blood gas analysis was not performed, as oxygen saturation remained stable and the patient did not demonstrate signs of severe hypoxemia or carbon dioxide retention. Non-invasive or invasive ventilatory support was not required throughout the disease course.

Supportive respiratory management focused primarily on bronchodilator therapy. Intravenous doxofylline was administered to relieve airway spasm, and intermittent nebulized salbutamol was used to improve airway patency and alleviate wheezing. These measures provided symptomatic relief but were insufficient to control the localized necrotizing endobronchial lesions.

Respiratory symptoms gradually improved following combined systemic anti-tuberculosis treatment and repeated bronchoscopic interventions, with normalization of respiratory rate and resolution of wheezing prior to discharge.

### Bronchoscopic findings and localization

Repeat bronchoscopies demonstrated necrotizing endobronchial lesions predominantly involving the distal trachea, right main bronchus, and right upper and middle lobe bronchi, with significant luminal narrowing and caseous debris.

Four bronchoscopic interventions were performed, including mechanical debridement and intraluminal administration of isoniazid, amikacin, and dexamethasone.

### Outcome and follow-up

Following combined systemic and bronchoscopic therapy, airway patency progressively improved. At 18-month follow-up, the child was asymptomatic, with normal growth and neurodevelopment. Chest CT demonstrated complete lung re-expansion with only residual calcified nodules.

## Discussion

This case illustrates how EBTB may initially be subtle and progress rapidly in infants, especially in the presence of immunodeficiency. *IKZF1* mutations impair lymphocyte development and interferon-mediated immunity, which are crucial for TB control. Co-infection with *P. jirovecii* further complicated diagnosis and management.

Bronchoscopy was indispensable, not only for diagnosis but also for repeated therapeutic intervention. Early and repeated intraluminal therapy likely prevented irreversible bronchial fibrosis and stenosis.

## Data Availability

The datasets presented in this study can be found in online repositories. The names of the repository/repositories and accession number(s) can be found in the article/Supplementary Material.
